# Using qualitative comparative analysis in a systematic review of a complex intervention

**DOI:** 10.1186/s13643-016-0256-y

**Published:** 2016-05-04

**Authors:** Leila Kahwati, Sara Jacobs, Heather Kane, Megan Lewis, Meera Viswanathan, Carol E. Golin

**Affiliations:** RTI International, 3040 E. Cornwallis Rd., Research Triangle Park, NC 27709 USA; Departments of Medicine and Health Behavior, University of North Carolina, Chapel Hill, NC USA

**Keywords:** Qualitative comparative analysis, Adherence, Configurational analyses, Systematic review methods

## Abstract

**Background:**

Systematic reviews evaluating complex interventions often encounter substantial clinical heterogeneity in intervention components and implementation features making synthesis challenging. Qualitative comparative analysis (QCA) is a non-probabilistic method that uses mathematical set theory to study complex phenomena; it has been proposed as a potential method to complement traditional evidence synthesis in reviews of complex interventions to identify key intervention components or implementation features that might explain effectiveness or ineffectiveness. The objective of this study was to describe our approach in detail and examine the suitability of using QCA within the context of a systematic review.

**Methods:**

We used data from a completed systematic review of behavioral interventions to improve medication adherence to conduct two substantive analyses using QCA. The first analysis sought to identify combinations of nine behavior change techniques/components (BCTs) found among effective interventions, and the second analysis sought to identify combinations of five implementation features (e.g., agent, target, mode, time span, exposure) found among effective interventions. For each substantive analysis, we reframed the review’s research questions to be designed for use with QCA, calibrated sets (i.e., transformed raw data into data used in analysis), and identified the necessary and/or sufficient combinations of BCTs and implementation features found in effective interventions.

**Results:**

Our application of QCA for each substantive analysis is described in detail. We extended the original review findings by identifying seven combinations of BCTs and four combinations of implementation features that were sufficient for improving adherence. We found reasonable alignment between several systematic review steps and processes used in QCA except that typical approaches to study abstraction for some intervention components and features did not support a robust calibration for QCA.

**Conclusions:**

QCA was suitable for use within a systematic review of medication adherence interventions and offered insights beyond the single dimension stratifications used in the original completed review. Future prospective use of QCA during a review is needed to determine the optimal way to efficiently integrate QCA into existing approaches to evidence synthesis of complex interventions.

**Electronic supplementary material:**

The online version of this article (doi:10.1186/s13643-016-0256-y) contains supplementary material, which is available to authorized users.

## Background

Systematic reviews evaluating complex or multicomponent interventions often encounter substantial clinical heterogeneity in intervention components, settings, and populations studied, which often contribute to heterogeneity of effect size. Complex interventions are those that include multiple components that often but do not necessarily interact with each other [1–4]. The UK Medical Research Council suggests that characteristics such as the number and difficulty of behaviors required by those delivering or receiving the intervention, the number and variability of targeted outcomes, and the degree of flexibility of tailoring of the intervention all contribute to an intervention’s complexity [5]. In addition to the number of components an intervention has, complexity can also refer to properties of the system in which an intervention is implemented, such as setting, number of actors involved, and intervention target characteristics [6, 7]. Further, an intervention may employ multiple and varied implementation strategies [7]. As a result of these myriad sources of potential variation, complex interventions with a common underlying purpose may differ quite substantially from each other in form or function when implemented.

Accordingly, systematic review investigators face substantial methodological challenges to synthesizing bodies of evidence comprised of complex interventions [7]. Estimating summary effects via quantitative synthesis is often not possible because of heterogeneity. Reviewers may ignore underlying variation by only addressing an overall question of effectiveness (e.g., do these types of interventions work?), or reviewers may stratify the synthesis based on one or more aspects of variation, such as a specific intervention component, outcome, population, or setting [7]. However, multicomponent interventions with interdependent components may not be suitable for separation into distinct components, and assumptions about linear and additive effects of multiple components may not be valid [8]. Methods that can systematically explore heterogeneity based on an assumption of causal complexity and that can provide an analytic link between heterogeneity and outcomes would offer an enhancement to current systematic review methods.

Qualitative comparative analysis (QCA) is a case-oriented method to study complex phenomena originating from the comparative social sciences [9]; it has been proposed as a potential method for synthesizing evidence within systematic reviews [7, 10]. QCA uses mathematical set theory, which is the branch of mathematical logic that studies the properties of sets, to examine set relationships between combinations of condition sets (cf., explanatory variables) present among cases and an outcome set (cf., dependent variable). QCA can be useful for identifying complex (i.e., non-linear, non-additive) causal patterns that variable-oriented methods may miss [9, 11, 12]. Applying QCA within the context of a systematic review may enhance review findings for policy-makers and practitioners by systematically evaluating sources of heterogeneity that influence the success (or failure) of an intervention using an approach that preserves each study’s unique combination of intervention components or other features. How to apply QCA within the context of a systematic review and the suitability of the method for this context is not definitively known because few actual applications exist [13, 14]. Based on our experience conducting systematic reviews and our experience using QCA in primary research applications, we postulated that using QCA could offer additional insights within a systematic review of a complex intervention beyond traditional synthesis.

In this paper, we describe using QCA within a systematic review and examine its suitability for use within this context. We used data from an Agency for Healthcare Quality and Research (AHRQ)-sponsored review of interventions to improve medication adherence that was recently completed by members of our study team (M.V., C.G.) [15, 16]. Medication adherence is a complex behavior with multiple determinants that vary among individuals [17]. Interventions to improve adherence often involve combinations of behavior change techniques (BCTs), such as interventions to improve self-efficacy or change attitudes. They often use different delivery modes (e.g., telephone vs. in-person) and agents (e.g., physicians, nurses, non-licensed staff) over various intervals of time and at different intensities. Further, interventions may be designed to influence patient adherence through interventions targeted at the practitioner or healthcare system level in addition to patient-directed components. We chose this review to use with QCA because the heterogeneity among interventions and outcomes seemed amenable to exploration through a configural lens and because we had access to all of the raw data and institutional knowledge associated with the review.

We turned to QCA because too much clinical heterogeneity had precluded a meta-analysis and meta-regression. Further, the completed review did not attempt mixed-treatment comparisons because of heterogeneity in the usual-care comparators [18]. However, all of the aforementioned approaches are correlational in nature, based on the assumption that one true distribution of effect exists and that trial-level covariates independently and additively contribute to variation from the true effect. QCA is not a substitute for these quantitative approaches to synthesis when they are appropriate, but these methods may rarely be appropriate for complex interventions because of the underlying assumptions upon which they are based. Thus, QCA offers a systematic approach to potentially unpacking intervention variability and relationship to an outcome when the phenomena under investigation can be characterized as complex.

## Methods

We conducted two substantive analyses using QCA using data that was collected as part of a completed review. The first analysis sought to identify which combinations of patient-directed BCTs used across the body of evidence were necessary and/or sufficient for improving medication adherence, and findings from this analysis are presented in detail in a companion paper in this issue [19]. The second analysis sought to identify which combinations of implementation features (e.g., agent, mode) used across the body of evidence were necessary and/or sufficient for improving medication adherence. In the present paper, we discuss the methodologic approach applied to both analyses and highlight the added value and challenges we identified through its application in a systematic review.

### Overview of QCA

Consistent with a case-oriented approach, QCA was originally developed for use with a small to medium number of cases (*N* = 10 to 50), allowing researchers to preserve the iterative nature of the data collection, analysis, and interpretation that stems from familiarity with the cases, a hallmark of qualitative research. More recently, QCA has been used for applications involving larger sample sizes [12]. Used within a systematic review context, each individual study within the review represents a case.

QCA preserves the holistic nature of each case throughout the analysis by not deconstructing the case into its component variables for analysis. Unlike variable-oriented methods that are based on probabilistic assumptions, QCA uses data from empiric cases to identify set relationships, which can be interpreted as relationships of “necessity” or “sufficiency” that often characterize causally complex phenomena. These relationships are depicted as a solution that uses Boolean operators, such as “AND,” “OR,” and “NOT,” to formulate verbal statements of the relationship between explanatory variables (i.e., conditions in QCA terminology) and an outcome. The solution generated by QCA is analogous to the expression of a correlational relationship among variables using a regression equation; though unlike probabilistic methods, solutions do not offer an estimate of precision, likelihood of finding results due to chance, nor can they be used for statistical hypothesis testing. A truth table is the analytic device used in QCA, and software is used to conduct most analyses [12, 20]. A detailed methodological description of QCA, a hypothetical example of an analysis, and a glossary of terms related to QCA is provided as supplementary online material (Additional file 1).

### Application of QCA to the completed review

Members of our study team (M.V., C.G.) conducted the completed review using methods associated with the AHRQ Effective Health Care Program (available at http://www.ncbi.nlm.nih.gov/books/NBK47095/). The completed review was limited to US studies in adults with chronic conditions, excluding patients with HIV/AIDS, severe mental illness, and substance abuse because these conditions often require specialized interventions not applicable to general medical populations [15, 16]. Of 4124 citations identified in the completed review, 758 full-text articles were screened for eligibility. Of the 67 low- or medium-risk of bias studies included, 62 were randomized clinical trials and five were observational studies. Included studies were conducted among patient populations with ten different clinical conditions. Seven studies included populations with more than one clinical condition. Study authors did not use consistent language or a standard taxonomy to describe intervention type; thus, the review team developed categories of intervention types. Examples included “education with behavioral support,” “health coaching,” “medication monitoring and reminders,” “shared decision-making or decision aids,” “case management,” and “collaborative care.” Because of heterogeneity of populations and intervention types, a quantitative synthesis was not possible. The primary organizing framework for the qualitative synthesis was clinical conditions (e.g., hypertension, diabetes). Within each of the ten clinical conditions, adherence outcomes were synthesized by intervention type. For example, a low strength of evidence grade for benefit was assigned for the use of case management interventions among patients with diabetes based on evidence from three RCTs. Overall, this approach resulted in 40 strata, each of which was assigned a strength of evidence grade based on the one to five studies falling within the stratum. The completed review’s analytic framework, key questions, and a summary of the results are provided as supplementary online material (Additional file 2). In brief, this review found the most consistent evidence for effectiveness across clinical conditions for interventions that included case management and educational interventions.

**Fig. 1:**
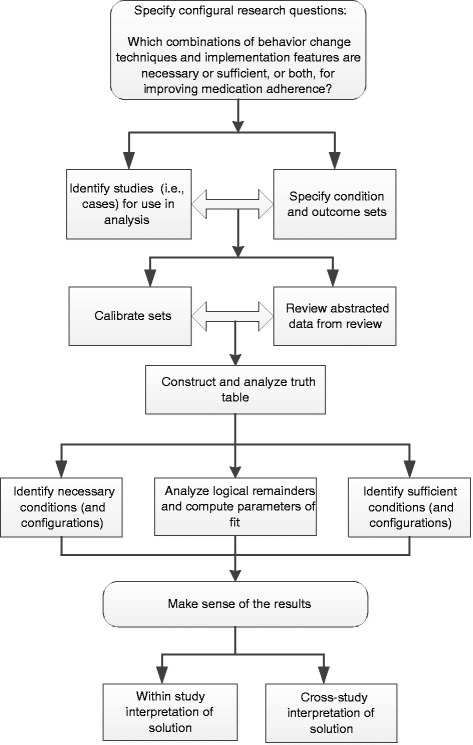
QCA approach used in this analysis. Adapted from Kane et al. [22]

We developed an approach to using QCA within the context of a systematic review based on existing standards of good practice for conducting QCA and our experience using the method in non-systematic review applications [21–23]. This approach is depicted in Fig. 1, and although the figure depicts this approach as sequential, in practice, iterative specification and analysis is typical and consistent with qualitative research approaches.

## Results

We will use the elements of Fig. 1 to summarize our process of using QCA with systematic review data.

### Specify configural research questions

As indicated in Fig. 1, we first specified a configural research question, which is a question designed to identify the combinations of conditions that produce an outcome. For each substantive analysis, we specified a single question that combined two of the completed review’s key questions. These were key question 1: “Among patients with chronic diseases with self-administered medication prescribed by a provider, what is the comparative effectiveness of interventions aimed at patients, providers, systems, and combinations of audiences in improving medication adherence?” and key question 3: “How do medication-adherence intervention characteristics vary?” Further, we specified both of the configural research questions to reflect causal asymmetry. The re-specified research question for the first QCA was “What combinations of behavioral change techniques are present in studies demonstrating improved medication adherence?” and for the second QCA was “What combinations of implementation features, such as agent, target, mode, span, and exposure are present in studies demonstrating improved medication adherence?”

### Identify studies for use in analysis

We defined studies included in the systematic review as the cases for each analysis. Based on how we operationalized the research questions, we excluded seven of the 67 studies from the completed review from both analyses as they were focused on policy or system level interventions and not relevant to the conditions (BCTs and implementation features) that we were interested in exploring. We found that the process used for study selection in a typical systematic review of interventions, which defines inclusion and exclusion criteria using the PICOTS framework (patient, intervention, comparator, outcome, timing, and setting), ensured that the cases included in the QCA were similar enough to be comparable, yet still offered enough diversity in intervention design to enable understanding heterogeneity of effect. Further, this approach provides an explicit and detailed rationale for the selection (or non-selection) of cases, which is a standard of good practice for conducting QCA [21].

### Specify and calibrate condition sets and outcome set

Because one of our study aims was to assess the suitability of using QCA in a systematic review context, we used a completed review to determine whether data typically abstracted during a review would be acceptable to use with QCA. Thus, our initial approach was to rely on the review’s completed data abstraction files and published evidence tables. However, we adjusted our approach during the course of the analyses to verify and supplement previously abstracted data as we needed additional information not collected during the original review process.

Set calibration refers to the process of assigning a numeric value between 0 and 1 based on data collected from or about the case for each condition set and outcome set included in an analysis. These values are referred to as set membership values and represent the degree to which the case belongs to each of the sets in the analysis. Researchers typically define the rubric that determines what set membership value to assign based on existing theory or information external to the cases at hand. Qualitative and/or quantitative data collected from a case is evaluated against the calibration rubric to determine the specific set membership value that should be assigned to the case. In a crisp-set (cf, binary) calibration scheme, cases are either assigned values of “1” (fully in the set) or “0” (fully out of the set). For example, when trying to establish whether an adherence intervention belongs to the set of studies that are “theory-based,” one could examine whether the intervention designers described and cited specific behavioral theories that were used to develop the intervention; if so, the study would be assigned a 1, and if not, the study would be assigned a 0. Non-binary calibration schemes are also possible and are described in more detail in the online supplementary material (Additional file 1).

Studies in the completed review used a variety of medication adherence outcomes measured at various time points based on self-report, prescription fills, or medication event monitoring systems (“smart” medication bottles). Some studies used more than one measure of adherence. We reviewed abstracted data and original studies and determined that we would consider studies to be fully in the set of studies with improved adherence if at least one measure of adherence demonstrated a statistically significant improvement as compared to a usual-care comparison group. We chose this calibration rubric because of the lack of a common adherence measure across studies. We considered using a fuzzy-set calibration rubric, which allows for set membership values between 0 and 1; but, the panoply of adherence measures used both within and across studies and the lack of external standards for defining differences in degree of adherence (e.g., “very much improved adherence” from “slightly improved adherence” from “slightly not improved adherence”) proved too challenging.

Condition sets used in each analysis are summarized in Table 1. The abstracted data and evidence tables that described the BCTs and implementation features used in studies generally provided inadequate information to enable us to calibrate condition sets; thus, we went back to original study publications to obtain more detail and to clarify ambiguous data abstraction entries for nearly all studies.

**Table 1:** Conditions sets used in two qualitative comparative analyses (QCA) within an existing systematic review of medication adherence interventions

Analysis 1 Behavior change techniques used^a^
Increasing knowledge—provision of general information about behavior-related health consequences, use of individualized information, increase in understanding/memory enhancement
Increasing awareness—risk communication, self-monitoring, reflective listening, behavioral feedback
Providing facilitation—ongoing professional support, dealing with adverse effects, individualizing/simplifying regimen (fewer pills, fewer medications, less frequent dosing, timing of dosing to fit individual schedule), reducing environmental barriers
Increasing self-efficacy—modeling, practice/skills training, verbal persuasion, coping response, graded tasks, reattribution of success/failure
Supporting intention formation—general intention, medication schedule, goals, behavioral contract
Increasing use of action control techniques—cues/reminders, self-persuasion, social support
Changing attitudes—targeting attitudes toward adherence behaviors
Supporting behavior maintenance—setting maintenance goals, relapse prevention
Using motivational interviewing—client-centered yet directive counseling style that facilitates behavior change through helping clients resolve ambivalence
Analysis 2 Implementation features
Intervention agent—the entity interacting with the intervention target to provide the intervention, for example health care professional, research assistant, automated computer or phone agent
Intervention target—the entity receiving the intervention, for example patient, provider, health care system, or combination
Span—the total length of time (in weeks) over which the intervention was provided
Mode of delivery—the mechanism through which the intervention was provided, for example in-person, over the phone, or virtually (online, text message, email, chat room, etc.)
Exposure—the total dose of the intervention (in minutes)

^a^A total of 12 behavioral change techniques were evaluated and abstracted during the completed review; we included these 9 in the QCA

The BCTs abstracted during the completed review were determined and defined a priori by the review team and derived from a previous meta-analysis of medication adherence interventions and a published taxonomy of BCTs [24, 25]. One study reviewer captured a study’s use of each BCT as “yes” or “no” or “unclear” based on information available in the published intervention description, and this was confirmed by a second reviewer. Thus, studies could be identified as using multiple BCTs. To studies that used a BCT, we assigned a set membership value of 1 for that BCT, and we assigned studies that did not use a BCT, or for which use of the BCT was unclear, a set membership value of 0. We also conducted sensitivity analyses with an alternate rubric that calibrated “unclear” as BCT use.

A challenge we encountered for the first analysis was the large number (12) of BCTs identified during abstraction in the completed review. With this many conditions, we were concerned about limited diversity that would result by including too many condition sets for the fixed number of studies (60). We winnowed the number of included condition sets to nine by eliminating three BCTs that were used by fewer than three studies. We attempted to further reduce the number of BCTs included in analysis by combining two BCTs to create a macrocondition, a typical strategy in QCA to reduce the number of included condition sets. However, we found the BCTs too conceptually distinct to combine into a single macrocondition. Thus, we could not implement a QCA standard of good practice with respect to keeping the number of condition sets relative to the number of cases at a reasonable level [21].

For the second analysis, which evaluated implementation features, we specified condition set-based implementation features that the completed review authors determined a priori and captured during study abstraction. These features, listed in Table 1, included intervention *agent*, *target*, *span* of intervention over time, *mode* of delivery, and intervention *exposure*. Information about these characteristics was captured by the review team using unstructured abstraction fields. For three of the condition sets, target, agent, and mode, the review team collapsed abstracted data into multivalue, mutually exclusive, categories for descriptive reporting of intervention characteristics.

We evaluated whether the multivalue categorical groupings for *target*, *agent*, and *mod*e could be further collapsed into dichotomous categories for a crisp-set calibration rubric. For target, the review team used information from the published description to assign each study to one of three categories: patient-only, combination of patient and provider, combination of patient and provider and system. For our analysis, we decided that the inclusion of a provider or system target, in addition to targeting the patient, was a key distinction as provider and system interventions would require additional training, infrastructure, and expense. Thus, we considered a study as “fully in” for the *target* condition set if the intervention targeted a provider or system in addition to a patient. Studies targeting only patients were considered “fully out” of the set. Similarly for *mode*, we first evaluated the completed review’s categorical groupings before deciding that a key design feature relevant to policy-makers and practitioners would be whether the intervention was delivered in-person versus some other mode (e.g., telephone, virtual, automated) because of secular trends in virtual care, convenience to patients, and perhaps lower costs. We developed two alternatives to accommodate interventions with mixed modes, where some of the intervention was delivered in person and some delivered by phone or virtually. For calibration of the *agent* condition set, we considered studies that used licensed health care professionals (e.g., nurse, physician, pharmacist) as fully in, and studies that used agents described as research assistants, health coaches, or other non-licensed types of staff as fully out.

The calibration of the final two condition sets in the second analysis, time *span* of intervention and intensity of *exposure*, exemplified the iterative back and forth between theory and empirical information from the cases at hand that is a QCA standard of good practice [21]. Study abstractors captured raw data about these two condition sets in an unstructured format during the review. We first transformed the raw data into standardized numeric values such that time span was represented in “weeks” from beginning to end of the intervention and the total time spent exposed to the intervention was represented in “minutes.” Because exposure information in some studies lacked detail, we made assumptions regarding average length of a clinic visit, telephone contact, or time spent exposed to an automated intervention when it was not specifically provided. For simplicity in interpretation, we chose to calibrate *span* and *exposure* with crisp sets. We contemplated various thresholds guided by the following considerations:

1. Select the calibration threshold with some knowledge of the range of values represented within our studies to avoid setting it too high or too low such that most studies would be in or out of the set.
2. Incorporate our substantive experience with behavioral interventions regarding what would be considered a threshold for a longer span or a higher exposure, but convey the condition sets using their numeric threshold value rather than terms such as low or high to mitigate concerns over the inherent arbitrariness of wherever we placed the threshold (e.g., span >12 weeks is “in,” rather than “long span” is “in”).
3. Test alternative thresholds in sensitivity analyses to assess the robustness of our findings with respect to the placement of the calibration threshold.

Ultimately, our main analysis used a calibration threshold of greater than or equal to 12 weeks as fully in the *span* condition set and a threshold of greater than or equal to 120 min as fully in the *exposure* condition set. In sensitivity analyses, we evaluated a *span* threshold of 6 weeks and two *exposure* thresholds, 60 and 240 min. We identified some differences in findings, and all supplemental analyses were made available as appendices to the main substantive analysis to support transparency and demonstrate the sensitivity of findings to changes in calibration thresholds.

### Construct and analyze the truth table

For each analysis, we transformed the raw data matrix of set membership values into a truth table, which places studies with the exact same configuration of set membership values for condition sets into the same truth table row. The number of logically possible truth table rows in an analysis is equal to 2^*k*^, where *k* is equal to the number of included condition sets; thus, the truth table for the first analysis contained 512 (i.e., 2^9^) rows and the table for the second analysis contained 32 rows (i.e., 2^5^). In both analyses, some of the truth table’s logically possible configurations were not present in any studies so these rows are “empty” of any empiric cases and are called logical remainders. The truth table is the analytic device in QCA for determining which configurations of condition sets consistently demonstrate the outcome. If all studies within a truth table row demonstrate improved adherence, then that row is coded as fully in or 1 with a consistency of 100 %. Rarely do real-world phenomena exhibit perfect consistency. In QCA, rows with a consistency of less than 100 % (also referred to as contradictory rows) can still be coded as 1 and included in sufficiency analyses if row consistency is above a prespecified level. Different thresholds for consistency can be used based on the nature of the research question, data quality, and number of cases, but typical thresholds are between 75 and 90 % [21].

Using the truth table created for each analysis, we identified set relationships between condition sets and configurations of condition sets and the outcome set. As described in the supplemental online materials (Additional file 1), superset relationships between condition sets and an outcome set can be interpreted as indicating necessary conditions. Similarly subset relationships between condition sets and an outcome set can be interpreted as indicating sufficient conditions. We used Stata Version 13 (StataCorp, College Station, TX) to create 2 × 2 contingency tables using set membership values for each condition set and the outcome set. Data from these tables are interpreted through a set-theoretic lens, meaning that the proportions produced by the table are interpreted as the consistency of each condition as a necessary condition for the outcome (% of cases in the outcome set that are also in the condition set) or as a sufficient condition for the outcome (% of cases in the condition set that are also in the outcome set). In the first analysis, we identified one BCT (techniques that increase knowledge) as individually necessary and one BCT (techniques that increase self-efficacy) as individually sufficient; in the second analysis, we did not identify any individually necessary or sufficient conditions.

Though an assessment of individually necessary or sufficient conditions is the initial analytic step, it is the evaluation of configurations of condition sets that allows QCA to offer powerful insights into complex causal patterns. For a configuration of condition sets to be necessary, it would need to be consistently present among all studies with the outcome of “improved medication adherence.” We did not identify two or more individual necessary condition sets in either analysis, and because formal logic prescribes that no configuration can be considered necessary unless each individual component condition set is necessary, we quickly discerned that we would not need an assessment of necessary configurations.

We used fsQCA version 2.5 to conduct sufficiency analyses for configurations [26]. In crisp-set QCA, the configuration of set membership values in each row of the truth table where the outcome set is 1 represents as expression of sufficiency. In other words, if the outcome is consistently present among cases within the row, then that unique combination of condition sets (i.e., presence or absence of conditions in a crisp-set scheme) is a sufficient pathway to the outcome. If multiple truth table rows consistently demonstrate the outcome, then multiple sufficient pathways are present (i.e., an equifinal solution). The most complex expressions of sufficiency can be taken directly from truth table rows; however, these statements are often unwieldy in the number of conditions and operator terms (ANDs, ORs, NOTs), which makes them difficult to interpret. These expressions can be logically minimized to simpler expressions with fewer terms and operators that are still logically consistent with the more complex expression, but easier to interpret.

The fsQCA software uses the Quine-McCluskey algorithm to perform this minimization procedure. The basis of this minimization procedure is that if two truth table rows with the same outcome differ in set membership value of only one condition set, then that condition set is irrelevant for producing the outcome in that row and can be eliminated. The two rows can be merged resulting in a simpler expression of sufficiency. This algorithm is repeated such that all truth table rows are compared and reduced until no further simplification is possible. In actuality, three variants of the minimization procedure are used to produce three variants of a solution, the conservative, the intermediate, and the parsimonious solutions. These three solutions are all logically consistent with each other but represent different degrees of parsimony and differ with respect to whether logical remainders are used as part of the minimization procedure.

Ultimately, we identified seven sufficient configurations in the intermediate solution for the first analysis and four sufficient configurations for the second analysis. A summary of these results is in Tables 2 and 3. We computed parameters of fit to describe how well the set relationships we identified deviate from a perfect set relationship (i.e., consistency) and how well the solutions identified explain the outcome across all empiric cases included (i.e., coverage). See the online supplementary materials (Additional file 1) for additional information regarding parameters of fit.

**Table 2:** Summary of findings from analysis 1 evaluating combinations of behavior change techniques used by effective adherence interventions

Combinations of behavior change techniques		Consistency^b^ (%)	Coverage Number of cases (raw^c^ (%)/unique^d^ (%))
Combination 1	Increasing knowledge AND enhancing self-efficacy	100	17 cases (50/44)
Combination 2	Using motivational interviewing AND not using facilitation	100	4 cases (12/6)
Combination 3	Enhancing self-efficacy AND using intention formation AND improving attitudes AND not increasing awareness	100	2 cases (6/0)
Combination 4	Using action control AND not increasing knowledge AND not using facilitation AND not using maintenance strategies	100	1 case (3/3)
Combination 5	Enhancing self-efficacy AND using intention formation AND improving attitudes AND not using facilitation AND not using maintenance strategies	100	2 cases (6/0)
Combination 6	Increasing knowledge AND using facilitation AND increasing awareness AND using intention formation AND using action control AND not using maintenance strategies	100	2 cases (6/6)
Combination 7	Increasing knowledge AND using facilitation AND improving attitude AND not increasing awareness	100	3 cases (9/9)
Solution^a^ (%)	–	100	76

^a^The solution is the conjunction of all combinations identified; solution coverage refers to the number of studies that include at least one of the identified combinations of behavioral change techniques. The solution coverage for intermediate solution was 76 %, which means 26 of the 34 studies were represented by one or more of the identified combinations, leaving 8 effective studies unexplained

^b^Consistency refers to the proportion of studies covered by sufficient combinations of behavioral change techniques that demonstrated improved adherence

^c^Raw coverage is the number of studies with the configuration of conditions (*N* varies by configuration) divided by the number of studies demonstrating improved medication adherence (*N* = 34). Studies may be covered by more than one configuration because of the logical minimization that occurs to generate more parsimonious combinations of conditions and condition complements. Raw coverage can vary between 0 and 100 %, with higher coverage representing more empirical relevance

^d^Unique coverage is the number of studies that are only covered by the configuration (*N* varies by configuration) divided by the number of studies demonstrating improved adherence (*N* = 34)

**Table 3:** Summary of findings from analysis 2 evaluating combinations of implementation features used by effective adherence interventions

Combinations of implementation features (agent, exposure, mode, time span, target)		Consistency^b^ (%)	Coverage Number of cases (raw^c^ (%)/unique^d^ (%))
Combination 1	Agent: uses staff other than licensed health care professionals AND Target: patients plus provider and/or systems	100	6 cases (18/12)
Combination 2	Agent: uses staff other than licensed health care professionals AND Exposure: less than 120 minutes AND Mode: no face to face component AND Time span: more than 12 weeks	88	8 cases (21/15)
Combination 3	Agent: licensed health care professionals AND Exposure: more than 120 minutes AND Mode: includes a face to face component AND Time span: less than 12 weeks	100	4 cases (12/12)
Combination 4	Agent: licensed health care professionals AND Exposure: more than 120 minutes AND Time span: more than 12 weeks AND Target: patients only	100	4 cases (12/12)
Solution^a^ (%)		95	56

^a^The solution is the conjunction of all combinations identified. Solution consistency refers to proportion of studies covered by sufficient combinations of behavioral change techniques demonstrated improved adherence. Solution coverage refers to the number of studies that include at least one of the identified combinations of implementation features. The solution coverage for intermediate solution was 56 %, which means 19 of the 34 studies were represented by one or more of the identified combinations, leaving 15 effective studies unexplained. Alternative models using different calibration thresholds for exposure were also conducted. Higher exposure threshold (>240 versus <240 min) resulted in slightly lower solution coverage (53 %) and consistency (90 %). Lower exposure threshold (>60 versus <60 min) resulted in higher solution coverage (68 %) and consistency (100 %)

^b^Consistency refers to proportion of studies covered by each combination of implementation features that demonstrated improved adherence

^c^Raw coverage is the number of studies with the configuration of conditions (*N* varies by configuration) divided by the number of studies demonstrating improved medication adherence (*N* = 34). Studies may be covered by more than one configuration because of the logical minimization that occurs to generate more parsimonious combinations of conditions and condition complements. Raw coverage can vary between 0 and 100 %, with higher coverage representing more empirical relevance

^d^Unique coverage is the number of studies that are only covered by the configuration (*N* varies by configuration) divided by the number of studies demonstrating improved adherence (*N* = 34)

### Make sense of the results

We examined the studies covered by configurations in the identified solutions to narratively describe how these solutions were represented within a study and across studies for each analysis. The process of relating solution findings back to the studies was instructive for identifying the need for adjustments in condition set calibration. This process also helped us to think beyond numeric coverage levels when considering the relevance of the various configurations to the outcome that we identified. For example, in the first analysis, we found configurations that included the absence of various BCTs to be less interpretable than configurations mostly characterized by the presence of BCTs since interventions are not typically designed to explicitly exclude a BCT. Similarly, the process of re-reviewing the studies in light of the solutions they exemplified allowed us to reconsider the relevance of the *knowledge* BCT condition set, which we had identified as individually necessary. This condition was present in 57 of the 60 studies we used for the QCA and was generally exhibited within studies as providing patients with information about their disease, the medication used to treat, and benefits and side effects of treatment. Thus, membership in the knowledge BCT set was heavily skewed, and knowledge would likely be a necessary condition of whatever outcome set we defined, a concept described by QCA experts as a “trivial” necessary condition [12]. Lastly, in keeping with standards of good QCA practice, we repeated all analyses for the set of studies (*N* = 26) not demonstrating improved adherence [19].

## Discussion

We used QCA within a systematic review to identify combinations of BCTs and combinations of implementation features found among effective medication adherence interventions. The 40 strength of evidence grades in the completed review provided readers with a synthesis of the magnitude and direction of effect for 40 small groups of studies, each group characterized by the same clinical condition and type of intervention [16]. The QCA results we identified complement the completed review findings by synthesizing across the boundaries of clinical condition and typology to identify combinations of BCTs and implementation features present among the entire set of effective interventions. The QCA findings are not a replacement for the findings in the completed review; rather, they provide additional insights based on configurational questions. Configurational questions are often not formulated as review key questions or the evidence is deemed insufficient to answer such questions for a variety of reasons—for example, lack of trials with direct comparisons of various different intervention features. Yet, “what is the recipe for effectiveness?” is often the information that practitioners and policy-makers want to know when complex interventions and their outcomes are heterogeneous.

We judged QCA to be suitable for use within systematic reviews based on the similarity of processes that are already part of a typical evidence synthesis. In Table 4, we provide our assessment of the alignment between systematic review and QCA steps, specifically the identification of studies/cases to include, data collection, study/case assessment, analysis, and presentation of findings. Our retrospective application of the method was inefficient, requiring re-review of the original studies at various steps in the process. However, a retrospective approach was invaluable for identifying challenges and steps that might be required beyond a typical review process in order to apply QCA. Although we identified alignment at a number of steps, how best to present findings within the review deserves further prospective evaluation.

**Table 4:** Alignment between typical systematic review processes and a QCA process

Research step	Systematic review process	QCA process	Alignment
Identification of cases to include	Formalized process involving a replicable literature search strategy and study inclusion/exclusion criteria defined by dimensions of population, intervention, comparator, outcomes, timing, and setting.	Non-mechanistic, researcher-directed process that selects cases that share enough background similarity yet offer heterogeneity with respect to explanatory conditions and the outcome.	Good.
Data collection	Information from included studies abstracted and put into structured evidence tables. Information abstracted typically includes study setting, population, intervention description, and outcome estimates.	No standard approach, varies by study, and dependent on the research question and nature of data being used.	Adequate, but could be inefficient if QCA is not planned from the start.
Study/case assessment	Risk of bias: based on researcher assessment of study design and study execution using standard assessment domains. Other elements: reviewer-assigned study attributes based on assessment of information in published study description or provided from authors in response to a query.	Calibration rubric guides process; process should be transparent and replicable based on rubric.	Not well aligned. Current review processes may need strengthening to support a robust calibration process.
Analysis	Qualitative synthesis—narrative summary with strength of evidence grade(s), sometimes stratified for large or diverse bodies of evidence. Quantitative synthesis—meta-analytic methods to produce summary effect estimates for direct and sometimes indirect comparisons. Meta-regression to explore heterogeneity of effect.	Configurational based on non-correlational analysis of set relationships. Includes components involving logical minimization of truth table but also narrative summary exploring cases identified in the solutions generated.	Requires separate steps but should be coordinated such that the analyses complement each other.
Presentation of findings	Typically involves text summary, along with supporting detailed evidence tables and figures supporting quantitative synthesis (e.g., forest plots, meta-regression figures) organized using the key questions of the review’s analytic framework.	Typically involves presentation of solutions using symbolic notation and Boolean operators in addition to narrative description of findings. In some cases, Venn diagrams, or *X*-*Y* plots can be used to convey findings.	Unclear. Need additional experience to determine appropriate way to present and integrate QCA findings in a typical evidence report.

The alignment between systematic review processes and QCA at the study/case assessment step deserves highlighting because of the importance of this step for fidelity to standards of good QCA practice [21]. The distinction between the abstraction tasks of transcribing information from studies into evidence tables and making judgments about the use of various BCTs or implementation features based on information in the studies was not well defined during the original review. Calibration of sets for QCA requires a clear rubric for making set membership value assignments and a mechanism for recording the rationale for the assignment, similar to the approach used for risk of bias assessments. Making set membership value assignments in tandem with data abstraction may be efficient; however, calibration rubrics cannot always be determined a priori, and the familiarity with studies gained through abstraction may be helpful for finalizing the rubric. Even the most robust calibration processes may not overcome the paucity of information about intervention components, implementation features available in published study reports. We believe this may be the biggest challenge to applying QCA and encountered this issue in both our substantive analyses. Ultimately, enough information about the study needs to be available to support the set membership value assignment, though sensitivity analyses could mitigate the impact of missing information.

We identified several other applications of QCA within systematic reviews. To date, all applications of QCA to systematic reviews have been published and presented in separate manuscripts, and not as part of the main evidence report. Using data from a subset of studies in a review of community engagement interventions for public health and health promotion, Thomas and Brunton et al. applied QCA to identify which combinations of community engagement methods directed toward pregnant or new mothers were effective for promoting breastfeeding [13, 27]. Although this study had limited diversity and low solution coverage, the investigators could derive additional meaning from the analysis that went beyond the initial qualitative synthesis. We agree with these authors’ assertions about the challenge of finding the right balance between parsimony and complexity when defining condition sets. Candy et al. used QCA with a completed Cochrane systematic review to explore relationships between what patients identify as important components of interventions to improve medication adherence for chronic clinical conditions with what components are actually represented within effective interventions [14]. The authors discuss the challenge with the selection and processing of data that is far removed from its primary source by the time it appears in a systematic review, a challenge we also acknowledge and had not previously encountered in our use of QCA within primary research studies. We concur with the observations of both study authors regarding the lack of intervention detail reported in primary studies limiting the robust application of QCA within a systematic review context.

Our experience is limited to conducting two analyses within the same completed systematic review. Whether QCA is feasible and adds value within reviews that include a smaller or larger numbers of studies or a review that includes many different outcomes or studies where interventions are complex but do not have easily discernible components is uncertain. The extent to which this method could be applied to other systematic reviews of complex interventions is determined by a number of factors, some based on requirements of the method itself. For example, variability in the outcome is essential to this method; we selected the medication adherence review to apply QCA in part because studies in the review included interventions with demonstrated effectiveness and interventions where effectiveness was not demonstrated. Lastly, our study did not evaluate how to present and integrate results from QCA within a traditional qualitative or quantitative review in a way that minimizes the need for an in-depth understanding of the method, yet provides enough transparency for readers to judge the validity and reliability of the findings.

We offer several recommendations for use of this method in systematic reviews. First, ensure some of the review research questions are configural and based on an a priori understanding of the phenomenon under evaluation. Reviews with fewer than ten studies may not be good candidates for QCA because no more than two to three condition sets can be accommodated without creating substantial limited diversity and patterns among condition sets may just as easily identified by “eye-balling.” Finally, we recommend initial calibration rubric design prior to study abstraction for efficiency, but teams should plan to re-specify and re-review studies if needed before making final calibration decisions.

## Conclusion

In conclusion, QCA offers systematic reviewers an additional tool for evidence synthesis in reviews of complex interventions. Further prospective use of the method during a review is needed to identify further areas for process alignment, method refinement, and how best to integrate and present results from a QCA into a typical evidence synthesis report.

## Additional files

Additional file 1: Detailed description, example, and glossary. This file provides a detailed description of the historical context of QCA, methodologic foundations, and walks through a hypothetical example analysis to illustrate key features of the methods. A glossary of terms is also included. (PDF 521 kb) Additional file 2: Completed review analytic framework, key questions, and summary of findings. This file provides the analytic framework, key questions, and a summary of findings from the completed AHRQ systematic review of interventions to improve medication adherence that was used to examine the suitability of using QCA in a review of a complex intervention. (PDF 156 kb)

## Abbreviations

AHRQ: Agency for Healthcare Research and Quality
BCT: behavioral change technique
HIV/AIDS: human immunodeficiency virus/acquired immunodeficiency syndrome
PICOTS: patient, intervention, comparator, outcome, timing, and setting
QCA: qualitative comparative analysis
RCT: randomized controlled trial
UK: United Kingdom
