# Supplementary material for: Using qualitative comparative analysis in a systematic review of a complex intervention
Source: Syst Rev. 2016 May 4;5:82. doi: 10.1186/s13643-016-0256-y (PMC4875617; doi:10.1186/s13643-016-0256-y)
Supplement: Additional file 1: — Detailed description, example, and glossary. This file provides a detailed description of the historical context of QCA, methodologic foundations, and walks through a hypothetical example analysis to illustrate key features of the methods. A glossary of terms is also included. (PDF 521 kb) [file 13643_2016_256_MOESM1_ESM.pdf]

Additional File 1

Detailed description, example, and glossary

Using qualitative comparative analysis in a systematic review of a complex intervention

Leila Kahwati, MD MPH[1,] Sara Jacobs, PhD[1], Heather Kane, PhD[1], Megan Lewis, PhD[1,] Meera Viswanathan, PhD[1], Carol E. Golin, MD[2]

[1] RTI International, Research Triangle Park, NC

[2] Departments of Medicine and Health Behavior, University of North Carolina, Chapel Hill, NC

Corresponding Author:
Leila Kahwati, MD MPH
RTI International
3040 E. Cornwallis Rd.
Research Triangle Park, NC 27709
Tel: (919) 541-6629
Fax: (9109) 541-6683
lkahwati@rti.org

CONTENTS

Introduction to Qualitative Comparative Analysis 1
Historical Context 1
Underlying Principles and Assumptions 1
Simplified Example 2
Set Relationships 2
Calibration and Truth Table Construction 3
Analysis of Necessity and Sufficiency 8
Glossary 12
Selected Sources for Additional Information 21
Website 21
Books 21
Journal Publications and Monographs 21
References 22

Introduction to Qualitative Comparative Analysis

Historical Context

Qualitative comparative analysis (QCA) is a case-oriented method originating from the comparative social sciences. Case-oriented methods and variable-oriented methods, such as regression, both have a role in research that advances understanding of effective health care interventions. Variable-oriented methods typically deconstruct the unit of analysis (e.g., patient, hospital, community, etc.) into its component variables and then assess statistical correlations among one or more variables.[1, 2] Variable-oriented methods excel for hypothesis testing and making population generalizations based on experiments or observations conducted on a random sample of the population. In contrast, case-oriented methods seek to examine or interpret theory through an in-depth examination within and across cases that share some degree of similarity. These methods focus on the case as a whole and examine the case's combinations of variables in relationship to the outcome. It is a useful approach for identifying complex causal patterns that variable-oriented methods may not find.[1-4] The developers of QCA believed that traditional variable-oriented statistical techniques were not well suited for explaining complex social phenomena, and an overall frustration with having to reformulate research questions to meet the requirements of statistical methods (such as large sample sizes) served as the impetus for developing this alternative approach.[3]

QCA is based on three foundational concepts: (1) John Stuart Mill's canons of experimental inquiry (method of agreement, method of difference, and joint method) and Boolean algebra, (2) a perspective on causation based on necessary and sufficient conditions, and (3) combinatorial explanatory models.[5] Although the set-theoretic comparative methods can be used for concept formation, creation of typologies, or causal inference, the term *qualitative comparative analysis* broadly describes a particular comparative approach and narrowly refers to a specific analytic technique that is most often used for causal inference.[6] Consistent with a case-oriented approach, QCA was originally developed for use with a small to medium number of cases (N=10 to 50), allowing researchers to preserve the iterative nature of the data collection, analysis, and interpretation that stems from familiarity with the cases, which is a hallmark of qualitative research. More recently, QCA has been used in for applications involving larger sample sizes. [6]

Underlying Principles and Assumptions

QCA has its own vocabulary and some readers may be unfamiliar with it. Consequently, we provide a glossary in this appendix. Throughout this introduction, we identify words that are defined in the glossary by bold italic font upon first use; for example, ***case***.

Similar to other case-oriented approaches, QCA seeks to preserve the holistic nature of each ***case*** throughout the analysis by not deconstructing the case into its component variables for analysis. In QCA, explanatory variables are called ***conditions***, and the dependent variable is called the ***outcome*** and both are operationalized as ***sets*** for analysis. The goal of QCA is to determine which conditions (or combinations of conditions) are ***necessar***y or ***sufficient***, or both, for the outcome based on the empirical data found in the cases. The concepts of necessity and sufficiency derive from formal logic and have precise meanings when applied to examining the relationship between conditions and an outcome.

QCA uses set theory, a branch of mathematical logic that studies the properties of sets, to examine relationships between combinations of conditions present among cases and an outcome. Unlike variable-oriented models, which assume linear additive, multiplicative, or logarithmic relationships between independent variables and a dependent variable, ***set-theoretic*** methods characterize the relationships between conditions and an outcome as set relationships. Set relationships identified using QCA are depicted as ***solutions*** that use ***Boolean*** operators, such as “AND”, “OR”, and “NOT”, to formulate verbal statements of necessity and sufficiency between conditions, combinations of conditions, and an outcome. The solution generated by QCA is analogous to the expression of a statistical relationship among variables via a regression equation. Three key concepts distinguish set-theoretic methods from variable-oriented methods: ***equifinality***, ***conjunctural causation***, and ***asymmetry of causation***. These three concepts make QCA particularly well suited for evaluating phenomena that are ***causally complex***.

Simplified Example

Set Relationships

The example in **Figure 1** illustrates how set relationships can be used to explore relationships between conditions and an outcome. In the analysis presented in the main study, conditions were operationalized to represent different behavior change techniques and the outcome was operationalized to represent improved medication adherence. In Figure A-1, each circle of the figure represents a set. Each of the A, B, C, D and X circles represents the set of cases with or without each of the five various intervention components, contextual features, or other characteristics of the population, setting, or intervention identified as conditions A, B, C, D, and X. The Y circle represents the set of cases that demonstrates the outcome of interest. Each number in the figure represents a unique case (e.g., program, study, clinic, hospital, community, etc.); case membership within each set is depicted by the location of the number within the circle. For example, Case 10 has individual set membership in Set D, Set A, Set Y, and Set X. A case located outside of a circle represents membership in the ***complement*** of the set. For example, Case 10 is in the complement of Set B and Set C. In addition to having membership in individual condition sets, cases have membership in sets that represent the conjunction of sets, sometimes referred to as combinations of conditions. For example, Case 1 has membership in the set that represents the conjunction of Set X, Set Y, Set B, Set C, Set D, and the complement of Set A. Similarly, Case 7 and Case 5 have membership in the set that represents the conjunction of Set A, Set B, and the complements of Set X, Set Y, Set C, and Set D.

Figure 1. Venn diagram of a simplified example depicting set relationships between 5 conditions and an outcome set using data from 20 cases

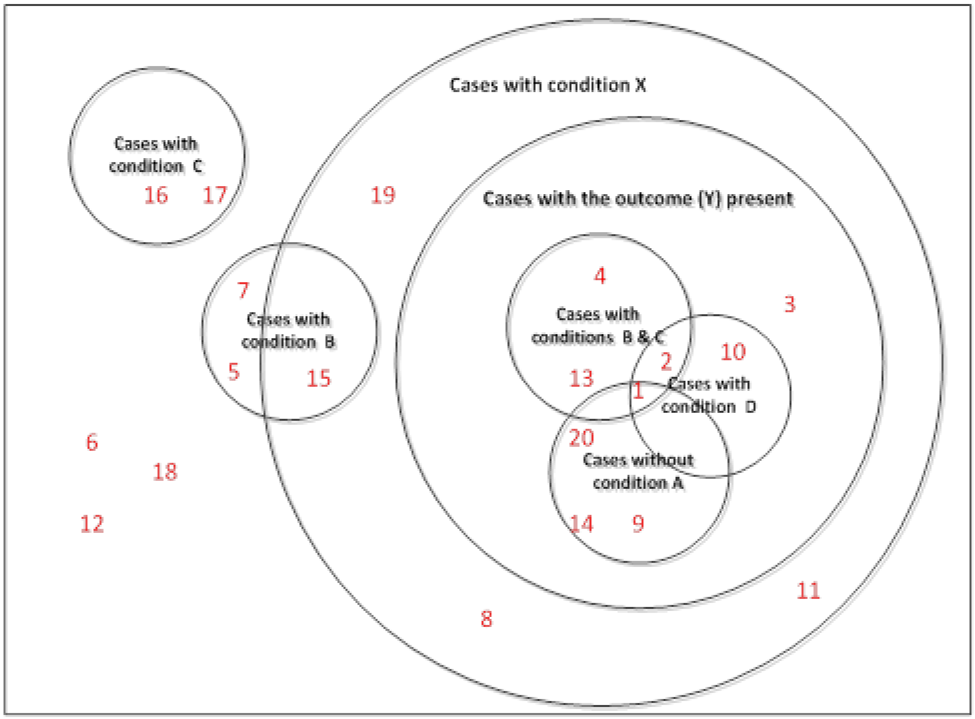

Condition sets (or sets representing combinations of conditions) are identified as necessary if the set of cases in the condition set exhibits a superset relationship to the set of cases that defines the outcome set. In plain language, a necessary condition is always present when the outcome is present. In Figure A-1, cases within condition Set X are a superset of the cases within the outcome Set Y. Cases within the Set Y must logically be in Set X. However, Set X is not sufficient for the outcome; a case can be in the condition Set X yet still be outside of the outcome Set Y as seen in cases 8, 11, 15, and 19 in Figure A-1. Thus, condition X is necessary but not sufficient for the outcome Y.

Sufficient conditions (or sets representing combinations of conditions) are subsets of an outcome set. In plain language, the outcome is always present when a sufficient condition is present. In Figure A-1, cases within the complement of condition Set A, or within condition Set D, or within the ***conjunction*** of condition Sets B and C are all subsets of the outcome Set Y. A case with any one of these sufficient conditions (NOT A, D, B AND C) is sufficient for being in the outcome set. Yet, a specific sufficient condition (NOT A, D, or B AND C) does not have to be present to be in the outcome set. Some cases may be in more than one sufficient condition set, as seen in cases 1 and 2 in Figure A-1. Similarly, some cases may be in the outcome set, yet lack any of the conditions specified in the analysis (e.g., case 3 in Figure A-1), suggesting that other sufficient conditions for the outcome may exist that were not included in the analysis.

Calibration and Truth Table Construction

Readers are referred to additional sources at the end of this appendix for discussion regarding the process of selecting cases, conditions, outcomes and ***set calibration***, including the optimum number of

cases and conditions to includes, and techniques for managing analytic problems such as ***limited diversity.*** We briefly summarize the process below to aid in understanding our main study methods and results using a simplified example of ***crisp- set*** QCA corresponding to ***Figure 1***.

Using qualitative data, quantitative data, or both from cases, each case is assigned a ***set membership value*** corresponding to its membership in each condition set. In this example, ***crisp-set calibration*** is used, and a "1" represents full set membership and a "0" represents full set nonmembership. For example, in the analysis presented in the main study, each study was assigned either a "1" or a "0" for each of nine possible behavior change techniques. Once each case has been assigned a set membership value for each condition, a data matrix with cases as rows and conditions as columns is generated that displays these data. An example of a data matrix corresponding to ***Figure 1*** is in ***Table 1***.

Adding a column with each case's outcome set membership value to the data matrix allows it to be used to analyze how individual conditions are either necessary or sufficient for the outcome. The assessment of individual necessity and sufficiency is analogous to process of conducting bivariate analyses prior to multivariate analysis; it helps the researcher get a "feel" for the data, but the findings related to individual conditions are generally of less interest as the configuration of set membership values across all conditions is the main unit of analysis, and the interpretation of the necessity or sufficiency of individual conditions does not offer any additional advantages over traditional correlational analyses.

For a condition to be individually necessary, it must consistently be present when the outcome is present. For a condition to be individually sufficient, the outcome must be consistently present when the condition is present. We use 2X2 contingency tables generated in Stata to assess necessity and sufficiency of individual conditions as seen in ***Figure 2*** and ***Figure 3***. We note that configural questions are constructed asymmetrically such that only some cells in the 2X2 contingency table are relevant to making a determination of necessity and sufficiency. For example, to evaluate the necessity of Condition A, we only need to assess the cells shaded in grey of ***Figure 2***. When the outcome is present, condition A is only present in 5 of 9 cases (55%), which is well below a consistency threshold of 90% typically used to define individual conditions as "necessary". To evaluate the sufficiency of Condition A, we only need to assess the cells shaded in grey of ***Figure 3***. When condition A is present, we find the outcome is present in 5 of 16 cases (31%), which is also well below standard consistency thresholds for sufficiency.

Next, the data matrix is converted into a ***truth table***, which has 2^k rows, where k is equal to the number of conditions included; in this example, five conditions results in 32 rows. The truth table combines cases with the exact same ***configuration*** of set membership values for conditions sets into the same row. **Table 2** demonstrates the cases from the data matrix that share the same configuration (identified by colors) that will be placed into the same truth table row.

Figure 2. Two-by-two contingency table used to evaluate necessity of individual conditions[a]

	Membership in Condition Set A (1)	Non-membership in condition Set A (0)	Total
Membership in Outcome Set (1)	5	4	9
Non-membership in Outcome Set (0)	11	0	11
Total	16	4	20

[a] In this table, only the shaded cells are of relevance to determining the necessity of Condition A for the Outcome.

Figure 3. Two-by-two contingency table used to evaluate sufficiency of individual conditions

	Membership in Condition Set A (1)	Non-membership in condition Set A (0)	Total
Membership in Outcome Set (1)	5	4	9
Non-membership in Outcome Set (0)	11	0	11
Total	16	4	20

[a] In this table, only the shaded cells are of relevance to determining the sufficiency of Condition A for the Outcome.

Table 1. Data Matrix Displaying the Set Membership Values for Each Case in Each Condition Set in the Example Analysis

	Condition Sets				
**Case ID**	**A**	**B**	**C**	**D**	**X**
1	0	1	1	1	1
2	1	1	1	1	1
3	1	0	0	0	1
4	1	1	1	0	1
5	1	1	0	0	0
6	1	0	0	0	0
7	1	1	0	0	0
8	1	0	0	0	1
9	0	0	0	0	1
10	1	0	0	1	1
11	1	0	0	0	1
12	1	0	0	0	0
13	1	1	1	0	1
14	0	0	0	0	1
15	1	1	0	0	1
16	1	0	1	0	0
17	1	0	1	0	0
18	1	0	0	0	0
19	1	0	0	0	1

	Condition Sets				
**Case ID**	**A**	**B**	**C**	**D**	**X**
20	0	0	0	0	1

Table 2. Data Matrix Displaying Cases That Share the Same Configuration; Rows with the Same Color Represent the Same Configuration

	Condition Sets				
**Case ID**	**A**	**B**	**C**	**D**	**X**
1	0	1	1	1	1
2	1	1	1	1	1
3	1	0	0	0	1
4	1	1	1	0	1
5	1	1	0	0	0
6	1	0	0	0	0
7	1	1	0	0	0
8	1	0	0	0	1
9	0	0	0	0	1
10	1	0	0	1	1
11	1	0	0	0	1
12	1	0	0	0	0
13	1	1	1	0	1
14	0	0	0	0	1
15	1	1	0	0	1
16	1	0	1	0	0
17	1	0	1	0	0
18	1	0	0	0	0
19	1	0	0	0	1
20	0	0	0	0	1

Table 3 is the truth table that corresponds to ***Figure 1*** and the data matrix in ***Table 1*** and ***Table 2***. This table has 32 rows that depict all of the logical possibilities of set membership values for five conditions. Notice that the cases in the analysis do not demonstrate configurations that cover all of the logically possible combinations. The rows that are "empty" of any empiric cases are referred to as ***logical remainders***.

The next step is to review the cases within each truth table row to assess whether the cases in each row consistently demonstrate membership in the outcome set. If so, the row is coded as having a set membership value of "1" in the outcome set. If cases within the same row differ on the outcome such that consistency is below a prespecified threshold, then the row is considered contradictory, and may or may not be used for further analysis. Different thresholds for ***consistency*** can be used, but a typical threshold is 80%. Row 7 in ***Table 3*** is an example of a contradictory row; one of the cases (case 3) is in the outcome set, whereas three cases (cases 8, 11, and 19) are not. The outcome consistency for row 7 is 25%, well below typical thresholds, leading to a decision to code the outcome set membership value for row 7 as "0".

Analysis of Necessity and Sufficiency

Next, software can be used to perform analyses of necessity and sufficiency. Various software packages are available, although no one package has all of the features needed to perform a rigorous QCA. Several reviews of QCA software are available.[7, 8] Several approaches to analyzing necessity are available, but the truth table is the analytic device used for sufficiency analysis.

Inspection of the truth table in ***Table 3*** demonstrates that in all cases where Y is present (rows 5, 9, 11, 23, and 25), X is present. A case cannot be in Set Y without also being in Set X; this type of set relationship is characterized as a superset relationship. This suggests that condition X is a necessary condition for the outcome Y. However, condition X is not sufficient for outcome Y as seen in rows 7 and 15. In these rows, cases are in condition Set X, yet they are not consistently in the outcome Set Y.

Each row of the truth table that consistently demonstrates membership in the outcome set (i.e., where Y set membership value is equal to "1") is itself a statement of sufficiency. For example, the configuration of set membership values for row 11 of ***Table 3*** (i.e., A(1) AND B(1) AND C(1) AND D(0) and X(1)) is a statement of sufficiency for the outcome Set Y because the cases represented by row 11 consistently exhibit set membership in the outcome Set Y. ***Table 4*** summarizes the five statements of sufficiency that can be gleaned from the truth table. The expression in the last column of ***Table 4*** uses symbolic notation to represent set membership values in the condition sets. An asterisk (*) is used to symbolize a logical "AND" and a tilde (~) is used to symbolize a logical "NOT," which is used to indicate a condition's complement (i.e., set membership value equal to 0). The symbol "→" in the expression represents an "if, then" relationship, which is a verbal way of expressing sufficiency. Some researchers and software packages use uppercase letters to express membership in the condition and lowercase letters to represent membership in the complement, and omit the AND and NOT operators. For example, the expression for row 11 may also be written as follows: ABCdX.

Table 3. Complete Truth Table

Row Number	A	B	C	D	X	Outcome Consistency	Outcome Set Membership Value	Case IDs
1	1	0	1	1	1	—	—	Logical remainder
2	1	0	1	1	0	—	—	Logical remainder
3	1	0	1	0	1	—	—	Logical remainder
4	1	0	1	0	0	0	0	16, 17
5	1	0	0	1	1	100%	1	10*
6	1	0	0	1	0	—	—	Logical remainder
7	1	0	0	0	1	25%	0	3, 8, 11, 19**
8	1	0	0	0	0	0	0	6, 12, 18
9	1	1	1	1	1	100%	1	2*
10	1	1	1	1	0	—	—	Logical remainder
11	1	1	1	0	1	100%	1	4, 13*
12	1	1	1	0	0	—	—	Logical remainder
13	1	1	0	1	1	—	—	Logical remainder
14	1	1	0	1	0	—	—	Logical remainder
15	1	1	0	0	1	0	0	15**
16	1	1	0	0	0	0	0	5, 7
17	0	0	1	1	1	—	—	Logical remainder
18	0	0	1	1	0	—	—	Logical remainder
19	0	0	1	0	1	—	—	Logical remainder
20	0	0	1	0	0	—	—	Logical remainder
21	0	0	0	1	1	—	—	Logical remainder
22	0	0	0	1	0	—	—	Logical remainder
23	0	0	0	0	1	100%	1	9, 14, 20*
24	0	0	0	0	0	—	—	Logical remainder
25	0	1	1	1	1	100%	1	1*
26	0	1	1	1	0	—	—	Logical remainder
27	0	1	1	0	1	—	—	Logical remainder
28	0	1	1	0	0	—	—	Logical remainder
29	0	1	0	1	1	—	—	Logical remainder
30	0	1	0	1	0	—	—	Logical remainder
31	0	1	0	0	1	—	—	Logical remainder
32	0	1	0	0	0	—	—	Logical remainder

* Indicates rows used to evaluate necessity, and for subsequent truth table analyses.
**Indicates rows where X is present, but outcome is absent, confirming that X may be necessary, but it is not sufficient for the outcome.

Table 4. Collapsed Truth Table Displaying Only the Five Rows That Demonstrate Sufficiency for the Outcome

Row Number	A	B	C	D	X	Y	Case IDs	Expression
5	1	0	0	1	1	1	10	A*~B*~C*D*X → Y
9	1	1	1	1	1	1	2	A*B*C*D*X → Y
11	1	1	1	0	1	1	4, 13	A*B*C*~D*X → Y
23	0	0	0	0	1	1	9, 14, 20	~A*~B*~C*~D*X → Y
25	0	1	1	1	1	1	1	~A*B*C*D*X → Y

Although the expressions in the last column of ***Table 4*** do represent statements of sufficiency, their complexity in terms of number of conditions and operator terms within each expression makes them difficult to interpret. These expressions can be ***logically minimized*** to simpler expressions that are still logically consistent with the more complex expression, but that are easier to interpret. Software is used to perform the logical minimization on these expressions using an algorithm called ***Quine-McCluskey***. Once simplified, each expression is referred to as a ***prime implicant. Table 5*** depicts the prime implicants resulting from logical minimization of ***Table 4***. The combination of all prime implicants with a logical "OR", also denoted using a "+" symbol, is called a ***solution***.

The solution in ***Table 5*** corresponding to this example exhibits the following characteristics:

- X is a necessary condition; it appears in each of the three sufficient prime implicants.
- The solution exhibits equifinality; three sufficient ***paths*** to the outcome exist.
- The solution has 100% consistency for sufficiency, all cases with any of the three prime implicant configurations are members of the outcome set. Had the contradictory row (row 7) from Table A-3 been included in the logical minimization process, the solution consistency would have been lower than 100%.
- The solution has 89% ***coverage*** (8 cases covered by at least one prime implicant divided by 9 cases in the outcome set). One case (Case ID 3) is not covered by any of the identified prime implicants.

Table 5. Three Prime Implicants and Overall Solution Derived from Logical Minimization of the Truth Table

Conditions	Simplified Expression	Verbal statement of sufficiency	Case IDs covered	Parameters of Fit*
		**Three Prime Implicants**		
Set X AND conjunction of Set B AND Set C	X*B*C → Y<br><br>Also written as: XBC→Y	The conditions X and B and C in combination are sufficient for the outcome Y.	1, 2, 4, 13	Consistency 100%<br>Raw coverage 44%<br>Unique coverage 22%
Set X AND complement of Set A	X*~A → Y<br><br>Also written as: X*a→Y or X~A→Y	The conditions X and the complement of A in combination are sufficient for the outcome Y.	1, 9, 14, 20	Consistency 100%<br>Raw coverage 44%<br>Unique coverage 33%
Set X AND Set D	X*D→Y<br><br>Also written as: XD→Y	The conditions X and D in combination are sufficient for the outcome Y.	1, 2, 10	Consistency 100%<br>Raw coverage 33%<br>Unique coverage 11%
		**Solution**		
Set X AND one of the following:<br>Set B AND Set C, OR<br>complement of Set A, OR Set D	X*(B*C+~A+D)→Y	The condition X in combination with B AND C, or not A, or D is sufficient for the outcome Y.	1, 2, 4, 9, 10, 13, 14, 20	Consistency 100%<br>Solution coverage 89%

*Parameters of fit refer to ***consistency*** and ***coverage***; they convey the extent to which the set relationships identified deviate from perfect set relationships.

In practice, several variations of the solution can be generated that are logically consistent with each other, but which represent different degrees of parsimony depending on whether logical remainders are included in the logical minimization process. See the entry for ***solution*** in the glossary for further detail.

The final analysis step is the process of relating the solutions identified back to the cases covered. In other words, using the cases to exemplify and explore the solutions identified. This can be done in a variety of ways. As part of this step, a discussion of deviant cases—cases that are responsible for lower consistency or that are not covered by any solutions identified—can also be explored. When relating back to cases, a focus on single conditions in a solution should be approached with caution in order to correctly convey its relevance, typically as a component of an overall configuration.

Glossary

asymmetric causality. An aspect of complex causality and set-theoretic methods that refers to the notion that the ***solution*** generated for the occurrence of an outcome does not automatically imply the solution for the ***complement*** (i.e., nonoccurrence) of the outcome. In other words, having a condition may produce the outcome, but this does not automatically mean that NOT having a condition will fail to produce the outcome. Most correlational methods assume symmetric causality, but this assumption may not be accurate for complex phenomena.

Boolean. Refers to combinatorial system of logic developed by George Boole that uses the operators AND, OR, and NOT to combine propositions, sets, or other entities (e.g., electric circuit states). Results of Boolean functions are expressed dichotomously as “True” or “False”, analogous to 1 and 0, using an analytic device called a ***truth table***. Boolean multiplication is equivalent to the logical operator “AND” or “*”, and Boolean addition is equivalent to the logical operator “OR” or “+”. The logical operator “NOT” or “~” is used to define a set ***complement***.

calibration. Refers to the process of assigning ***condition*** and ***outcome set membership values*** for each ***case*** that represents the degree to which the case satisfies membership criteria in the ***sets*** being used for analysis. Calibration requires relying on substantive knowledge or information about the case relative to an empirically or theoretically grounded referent. This knowledge may come from qualitative and/or quantitative data about the case. ***Crisp set*** and ***fuzzy set*** are two types of calibration used in ***qualitative comparative analysis***.

QCA relies on ***set membership values*** assigned through a process of calibration, not on measured values of a condition. The distinction between measurement and calibration is typified by an example involving blood pressure, which is measured in millimeters of mercury (mmHg). Calibration refers to establishing set boundaries based on externally determined thresholds that represent qualitatively different states; for example, elevated blood pressure (> 140/90mm Hg) or not elevated blood pressure (< 140/90 mm Hg). The calibration thresholds in this example derive from diagnostic thresholds for hypertension.

case. Similar to usage in qualitative research, the term *case* refers to the unit of observation and analysis. Cases can be individuals, organizations (e.g., schools, health care systems, churches, communities, networks, etc.), cultures, geopolitical units (e.g., counties, states, territories, countries, etc.), defined events, or any instance of something (e.g., scientific trial, bill/law, practice guideline).

causal complexity. Phenomena that are characterized by ***equifinality***, ***conjunctural causality***, and ***asymmetric causality*** are considered causally complex. ***Qualitative comparative analysis*** is a method that accommodates such features; consequently, it can be useful for exploring and understanding social, behavioral, and biological phenomena that exhibit causal complexity.

complement. Sometimes referred to as logical complement. Represents the ***set*** that contains all ***cases*** that are not members of an original set. For example, the complement of a set that contains cases that have an outcome present is the set of cases that do not have the outcome present. The complement is sometimes referred to as the negation of a set, and is expressed using the logical operator “NOT”.

condition (set). Conditions are the explanatory factors used in ***qualitative comparative analysis*** to understand and explain an ***outcome***. Conditions are analogous to independent variables (i.e., “X” variables) in probabilistic, correlational methods. Conditions are operationalized as condition ***sets*** and are combined with other condition sets and condition ***complement*** sets to create more complex sets.

configuration. The ***conjunction*** of ***set membership values*** for all ***conditions*** in an analysis that describes a unique combination of conditions. Each row of a ***truth table*** represents a theoretically possible configuration within a ***logical property space*** of 2^k configurations, where k is equal to the number of conditions. For example, an analysis with 3 conditions has 8 possible configurations in a truth table,

and an analysis with 5 conditions has 32 possible configurations. ***Cases*** in an analysis will exhibit one and only one configuration based on its set membership values and therefore will be covered by only one truth table row. Cases can have the same configuration and share the same truth table row if they have the same set membership value for all conditions. Some configurations are theoretical; that is, no empiric cases in an analysis demonstrate the specific combination of conditions. These configurations are also known as ***logical remainders***.

conjunction*. **Analogous to the logical operator *"AND"* and in set theory is sometimes referred to as an intersection. It refers to the combination of two or more sets such that the conjunction represents the set of ***cases that are in both of the individual condition sets. In other words, if a case is not in Set B, then it cannot be in the set that represents cases that are both in Set A AND Set B. The conjunction of ***set membership values*** for all conditions in a case represents that case's ***configuration***.

conjunctural causality*.** Is a feature of ***causal complexity. Refers to phenomena where the causal effect of an individual ***condition*** may only be exhibited in combination (i.e., ***conjunction***) with other specified conditions.

consistency*.** One of the ***parameters of fit calculated for individual ***conditions, prime implicants*** (i.e., complex sets representing ***conjunctions*** of multiple conditions and condition complements)**,** and overall ***solutions***. Most social, behavioral, and biologic phenomena rarely exhibit perfect superset and subset relationships; consequently, consistency is a way to numerically express the degree to which the empirical data from ***cases*** deviate from a postulated ***set relationship***. Consistency is a numeric value between 0 and 100% and is calculated separately for evaluating ***sufficiency*** and ***necessity***.

Consistency for sufficiency (crisp sets):

(# of cases in condition set and outcome set/# of cases in condition set) X 100

A set with 100% consistency for sufficiency would have a perfect subset relationship to the ***outcome*** set. That is, all of the cases in the condition set are also members of the outcome set. **Figure G-1** displays the concept of consistency of sufficiency for single conditions using Venn diagrams.

Figure G-1. Consistency of sufficiency for single conditions.

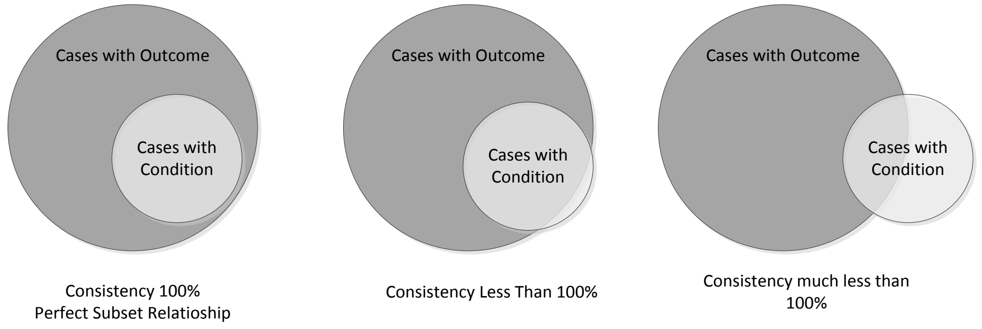

Low consistency for sufficiency indicates that many cases that exhibit a condition are not members of the set of cases that exhibit the outcome. High consistency for sufficiency indicates that many cases that exhibit a causal condition are also members of the set of cases that exhibit the outcome. Consistency for sufficiency of prime implicants (i.e., complex sets) is calculated similarly. The threshold used to determine sufficiency consistency is dependent on the nature of the research. Although 80% is a typically used threshold, higher thresholds are often used when there is a strong underlying basis for a relationship from existing evidence, high confidence in the ***calibration*** procedure used, or when a small number of cases is used. In larger N applications, probabilistic methods can be used to determine consistency thresholds.

Consistency for necessity (crisp sets):

(# of cases in condition set and outcome set/# of cases in outcome set) X 100

A condition with 100% consistency for necessity would have a perfect superset relationship to the outcome set. That is, all of the cases in the outcome set are also members of the condition set. **Figure G-2** displays the concept of consistency for necessity of single conditions using Venn diagrams.

Figure G-2. Consistency for necessity of single conditions.

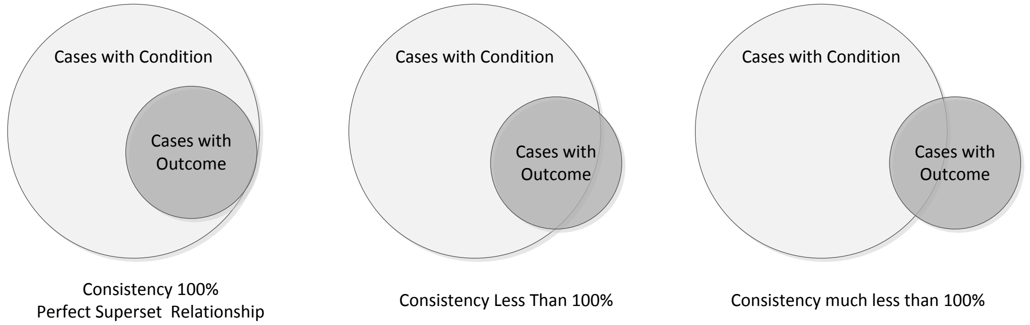

Low consistency for necessity means that many cases that exhibit the outcome are not members of the set of cases that exhibit the condition. High consistency for necessity means that many cases that exhibit the outcome are also members of the set of cases that exhibit the condition. Consistency for necessity of prime implicants (i.e., complex sets) is calculated similarly, although it is unusual to identify necessary prime implicants that comprise more than one individual condition in real-world application because in order for a prime implicant to be necessary, all conditions within the prime implicant expression must themselves be individually necessary. The threshold used to determine necessity consistency is also very much dependent on the nature of the research, although higher thresholds (> 90%) are typically used, as compared with sufficiency thresholds.

contradictory truth table rows. With crisp sets, these are ***truth table*** rows that contain cases with different set membership in the ***outcome*** set. In other words, these rows contain cases that share the same ***set membership values*** on all ***conditions*** but differ on the outcome. With fuzzy sets, contradictions are determined differently.

coverage. One of the ***parameters of fit*** calculated for individual ***conditions, prime implicants*** (i.e., complex sets representing multiple conditions and condition complements)**,** and overall ***solutions***. Coverage is only relevant for conditions and prime implicants that have already passed the threshold for ***consistency***. With respect to individual conditions, coverage expresses the proportion of the ***cases*** in an ***outcome*** set that are also members of a ***sufficient condition*** set. For prime implicants, coverage expresses the proportion of cases in an outcome set that are also members of a sufficient prime implicant (i.e., complex set).

Figure G-3 contrasts conditions with high and low coverage. Low coverage indicates that the cases that exhibit a sufficient condition (or prime implicant) do not represent a large proportion of the cases that exhibit the outcome. High coverage indicates that the cases that exhibit a sufficient condition (or prime implicant) do represent a large proportion of the cases that exhibit the outcome. In general, the higher the coverage, the more empirically relevant that condition may be as an explanation for the outcome. Conditions with low coverage may be sufficient, but the low coverage suggests that the condition (or prime implicant) may not be as commonly observed for the phenomena of interest, in contrast to those with higher coverage. In other words, conditions with low coverage may be trivial,

although they may still be of interest as an alternative explanation for an outcome, consistent with the principle of ***equifinality***.

Figure G-3. Contrasting conditions with high and low coverage.

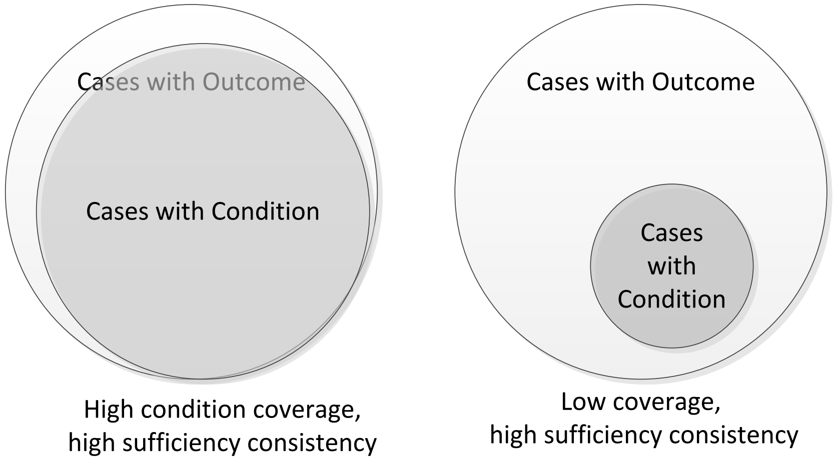

Raw coverage typically refers to prime implicant coverage. It represents the percentage of cases in the outcome set that are covered by a specific prime implicant that is part of a solution. Although each case is only covered by one ***truth table*** row, through ***logical minimization***, each case may end up covered by more than one prime implicant, because a prime implicant is a logically consistent but simpler expression of two or more truth table rows. Prime implicant coverage is calculated as follows for crisp sets:

(# of cases covered by a specific prime implicant/# of cases in outcome set) X 100

Unique coverage also refers to prime implicant coverage. It represents the percentage of cases in the outcome set that are uniquely covered by a specific prime implicant. In other words, it represents the proportion of cases that are only covered by a specific prime implicant. It is calculated as follows for crisp sets:

(# of cases uniquely covered by a specific prime implicant/# of cases in outcome set) X 100

Solution coverage refers to the percentage of all cases in the outcome set that are covered by at least one of the prime implicants in an equifinal solution. 100% solution coverage means that all cases in the outcome set are covered by at least one of the prime implicants identified. It is calculated as follows for crisp sets:

(# of cases covered by one or more prime implicants/# of cases in outcome set) X 100

Figure G-4 contrasts high and low solution coverage.

Figure G-4. Contrasting high and low solution coverage.

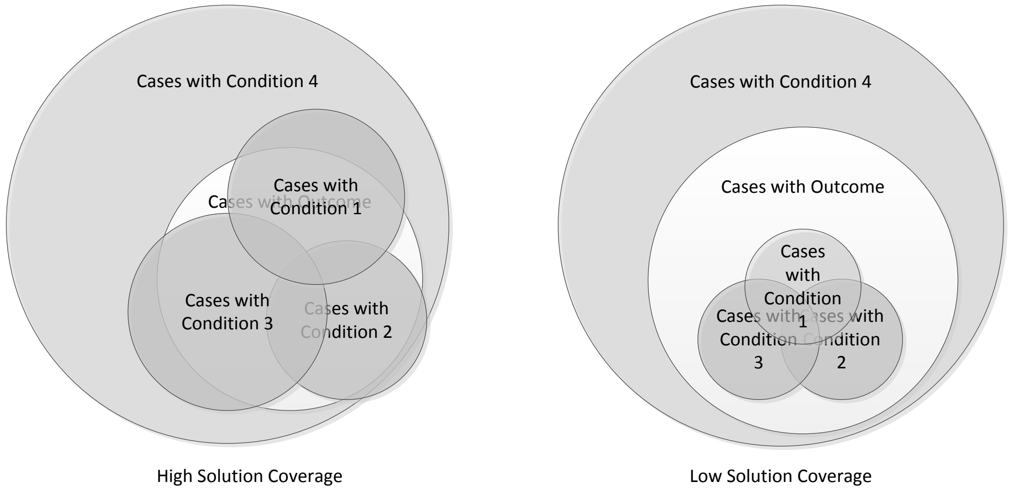

crisp-set calibration. Process of using qualitative and/or quantitative data or information from cases about a ***condition*** (or ***outcome***) and representing this information as a ***set membership value***. For crisp-set calibration, conditions are represented dichotomously, which allows only for full membership (“fully in” or “1”) and full nonmembership (“fully out” or “0”) in a set. For some conditions, fully in means the condition was simply present and fully out means the condition was absent. For other conditions that are not so easily characterized as simply present or absent, crisp sets can be calibrated such that fully in may mean that a case exhibits high levels, robust evidence, strong indications, or is above some threshold (preferably defined by an external standard or referent), whereas fully out means that cases that do not exhibit the criteria established set membership. Consequently, crisp sets are designed to reflect a qualitative distinction between those cases that are in the set and those cases that are not in the set. Alternatives to crisp-set calibration are ***fuzzy-set calibration*** or multivalue calibration.

crisp-set QCA (csQCA). A version of qualitative comparative analysis that uses crisp sets.

equifinality. A principle of complex social or biologic systems whereby the same outcome can be obtained with different causal mechanisms and/or starting conditions. QCA embodies this principle by being able to express multiple ***paths*** to an outcome. An equifinal solution is one characterized by having more than one ***prime implicant***.

fuzzy-set calibration. Process of using qualitative and/or quantitative data or information about a ***condition*** (or ***outcome***) from ***cases*** and representing this information in terms of a ***set membership value***. In addition to full membership (“fully in” or “1”) and full nonmembership (“fully out” or “0”), fuzzy-set calibration allows for set membership values between 0 and 1 to characterize partial set membership. This scheme allows for the representation of quantitative differences in degree of set membership among qualitatively similar cases. Under a ***crisp-set*** scheme, two cases with a condition present would have the same set membership value even if there were differences in the degree to which the condition was present. With fuzzy-set calibration, cases can take on values between 0 and 1 that represent the degree to which the condition is present (e.g., very high levels, high levels, medium high levels, etc.). A set membership value of 0.5 for fuzzy set represents the crossover point; fuzzy-set membership values above 0.5 represent a case that is more “in” than “out”, and values below 0.5 represent a case that is more “out” than “in”. As in crisp-set calibration, qualitative and quantitative thresholds for ***calibration*** should preferably rely on external standards or referents.

fuzzy-set QCA (fsQCA). A version of ***qualitative comparative analysis*** that uses ***sets*** that have been calibrated using ***fuzzy-set calibration***. May also be used to refer the software package designed to analyze crisp or fuzzy sets (fsQCA version 2.5).

limited diversity. Refers to analyses in which the empiric ***cases*** available do not adequately cover the ***logical property space*** available; in other words, the resulting ***truth table*** contains many ***logical remainders***. Limited diversity can result from not having enough cases relative to the number of truth table rows (which is determined by the number of included conditions). Alternatively, cases may cluster into just a handful of available ***configuration***s, either because cases may not naturally vary across the entire spectrum of included conditions; or because some of the configurations simply cannot exist in reality, as they would result in phenomena that are biologically or socially impossible (e.g., Set A = pregnant, Set B = man, the combination of pregnant and man is not possible). When limited diversity is present, the three ***solutions*** generated may differ depending on the assumptions made about the logical remainders.

logical minimization. The process of using the ***Quine-McCluskey algorithm*** to reformulate the ***primitive expressions*** of ***sufficiency*** from each ***truth table*** row into logically consistent but simpler terms, known as ***prime implicants***. The logical minimization process results in a ***solution***. The process of logical minimization can produce three different solutions depending on how ***logical remainders*** are handled during the process.

logical remainder. Refers to ***truth table*** rows in an analysis that are logically possible, but for which no empiric ***cases*** exist. For crisp sets or small to medium N-sized applications, this usually refers to truth table rows that do not have at least one case. Simplifying assumptions about ***logical remainders*** are made during the ***logical minimization*** process to result in a ***solution*** that is more parsimonious.

necessary condition or necessity. For ***crisp sets***, a ***condition*** is deemed necessary if the data analyzed demonstrate that the condition exhibits a superset relationship to the ***outcome*** set. ***Cases*** that are members of the outcome set must logically be members of a condition set. **Figure G-5** represents a necessary condition; a case that is in the outcome set must logically be in the condition set. In other words, if the data from an analysis show that when the outcome is present the condition is consistently present, then that condition can be considered necessary. Cases may be members of a necessary condition set without being members of the outcome set (i.e., light shaded area of Figure G-5). In other words, a case can have a necessary condition without having the outcome. A condition that is perfectly necessary and ***sufficient*** is one in which the ***conjunction*** of the condition set and the outcome set is equivalent to either set alone (i.e., perfect overlap of the sets in Figure G-5). For ***fuzzy sets***, a necessary condition is one for which set membership in the condition is larger than or equal to set membership in the outcome set, across all cases.

Figure G-5. A necessary condition.

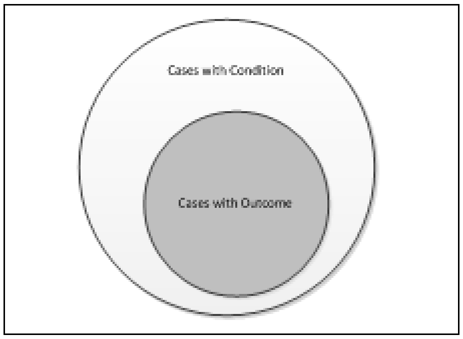

outcome (set). The dependent phenomenon that is evaluated in an analysis. An outcome set is ***calibrated*** to define boundaries for set membership based on the degree to which a case exhibits the outcome of interest. Cases that exhibit the outcome are assigned ***set membership values*** consistent with their degree of set membership. For example, a case that exhibits the outcome of interest using ***crisp sets*** is assigned a set membership value of "1", whereas a case without the outcome of interest is assigned a set membership value of "0".

parameters of fit. Numeric values that describe the extent to which the ***solution*** identified deviates from perfect ***set relationships***, and the extent to which the solution identified has empirical importance and relevance. Common parameters of fit include ***consistency*** and ***coverage***; these parameters can be

expressed at the level of a single ***condition***, ***primitive expression***, ***prime implicant***, or for the overall solution.

path. A terms that is sometimes used to refer to the combination of multiple ***conditions and/or condition complements*** that is sufficient for an outcome. A synonym for ***prime implicant***. It is often used in the context of describing ***equifinality***, or multiple "paths" to an outcome.

prime implicants. Expressions that result from application of the ***Quine-McClusky algorithm*** (i.e., ***logical minimization***) to a ***truth table***. These expressions result from pairwise comparison of ***primitive expressions*** (i.e., ***sufficient truth table*** rows) that differ in only one ***condition*** to see whether the expressions lead to the same ***outcome***. If the same outcome is present, then the differing condition is considered logically redundant and eliminated from the expression, creating a simpler expression. The process is repeated until no further minimization is possible and the remaining expression is termed a prime implicant. Prime implicant expressions can comprise single conditions; but typically are more complex sets involving ***conjunctions*** of multiple conditions and/or condition ***complements***. Multiple prime implicants combined with a logical "OR" comprise a ***solution***. Cases that have set membership in more than one prime implicant are said to be "covered" by more than one sufficient *path* (see ***coverage***). Cases that have set membership in only one prime implicant are said to be uniquely covered.

primitive expression. A term used to describe a ***truth table*** row that is sufficient for the ***outcome***. In other words, a row where empiric cases consistently demonstrate membership in the outcome set. In QCA, the ***logical minimization*** procedure uses the ***Quine-McCluskey algorithm*** to reformulate these primitive expressions into logically consistent but simpler terms, known as prime implicants.

qualitative comparative analysis (QCA). A set-theoretic method for cross-case analysis that uses formal logic and ***truth tables.*** Several variations of QCA exist including ***crisp-set QCA***, ***fuzzy-set QCA***, multivalue QCA, and temporal QCA.

Quine-McCluskey algorithm. Also referred to as the method of ***prime implicants***, or ***Boolean*** simplification. It is an algorithm for minimizing Boolean functions. In ***qualitative comparative analysis***, each ***truth table*** row represents a Boolean function, as each row is an expression of ***conjunctions*** of ***condition set membership values***. This algorithm is used for the ***logical minimization*** of truth tables and is a primary underlying feature of software designed for these analyses. The algorithm involves the pairwise comparison of ***primitive expressions*** (i.e., ***sufficient truth table*** rows) that differ in only one condition to see whether the same ***outcome*** is seen. If so, then the differing condition is considered logically redundant and eliminated from the expression, creating a simpler expression. The process is repeated until no further minimization is possible and the remaining expression is termed a prime implicant.

set. A mathematical term that refers to a well-defined or bounded collection of distinct objects (also referred to as set elements or members). Each object within a set is a distinct entity in and of itself; yet, the set that defines a collection is also considered a distinct entity upon which mathematical operators and functions can be applied. In a ***qualitative comparative analysis,*** a set is created for every ***condition*** and the ***outcome*** in the analysis***. Cases*** are considered the objects of the set, and are placed into condition and outcome sets through the process of ***calibration***. For example, the outcome set comprises all of the cases that have the outcome present, based on whatever set boundaries are established for determining the "presence of the outcome."

set-membership value. A numeric value that expresses the extent to which a case is a member of a set. ***Crisp-set*** membership values have only two values, full membership, expressed as a "1" (fully in) and full nonmembership, expressed as a "0" (fully out). ***Fuzzy-set*** membership values express degrees of membership in a set; for example, fully in, more in than out, more out than in, fully out. Fuzzy-set membership takes on values spanning from 0 to 1; with values above or below a qualitative anchor

(typically 0.5) denoting a qualitative difference in set membership, yet allowing for cases on the same side of the qualitative anchor to differ in their degree of set membership either continuously, or at designated quantitative intervals.

set relationships. See ***set-theoretic methods***.

set-theoretic methods. Qualitative comparative analysis is a specific type of a set-theoretic method. Set-theoretic methods refer to approaches that analyze phenomena through the use of ***sets*** and their relationships, in contrast to approaches based on statistical relationships among variables. Set-theoretic methods are distinguished by use of numeric values to describe a case's membership within a set and interpret set relationships in terms of ***sufficiency*** and ***necessity***. Set-theoretic methods are particularly useful for exploring ***causal complexity***.

solution. The result of the ***logical minimization*** of ***primitive expressions*** from sufficient ***truth table*** rows. A solution is the representation of the set relationships between ***conjunctions*** of ***conditions*** and an ***outcome***. A solution may include several ***prime implicants*** combined with a logical "OR", characterizing the principle of ***equifinality***. Solutions use symbols to describe "if, then" relationships between conditions and outcome (→) rather than symbols indicating equality ("="), which characterizes the ***causal asymmetry*** inherent in many phenomena studied using ***qualitative comparative analysis***.

The process of logical minimization can result in three solutions, which are logically consistent with each other but represent different degrees of parsimony.

The conservative solution (sometimes referred to as the complex solution) is based on no simplifying assumptions on ***logical remainders*** and usually results in the solution with the most number of conditions and operators (i.e., AND, NOT, OR).

The parsimonious solution is a superset of the conservative solution and is based on algorithm-driven simplifying assumptions on logical remainders that maximize parsimony to produce an expression with the fewest number of conditions and operators. However, some of the simplifying assumptions made to achieve parsimony may not be consistent with theoretic or empiric knowledge about a phenomenon, and therefore may be considered inappropriate to make.

The intermediate solution is a superset of the conservative solution, but a subset of the parsimonious solution. It is based on researcher-directed simplifying assumptions on logical remainders, such that the assumptions are consistent with directional expectations and untenable assumptions are avoided. This typically results in a solution that has more conditions and operators than the parsimonious solution, but not as many as the conservative solution.

Figure G-6. A necessary condition.

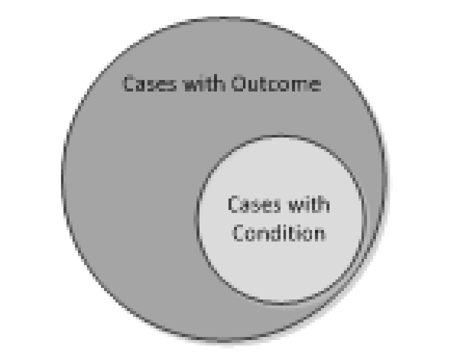

sufficient condition or sufficiency. For ***crisp sets***, a ***condition*** is deemed sufficient if the data analyzed demonstrate that the condition exhibits a subset relationship to the ***outcome*** set. **Figure G-6** represents a sufficient condition; ***cases*** that are members of a sufficient condition set must logically be members of the outcome set. In other words, if the data from an analysis show that when the condition is present, the outcome is consistently present, than that condition can be considered sufficient. Cases may be members of the outcome set without being members of a sufficient condition set (i.e., dark shaded area of Figure G-6). Complex phenomena often exhibit multiple sufficient conditions (i.e., ***equifinality***), and a case can have membership in more than one sufficient condition. Cases in the outcome set that are not members of any sufficient condition sets (i.e., a ***solution*** with low ***coverage***) suggest that there may be paths to the outcome that involve conditions

not included in the analysis. For ***fuzzy sets***, a sufficient condition exhibits set membership in the condition that is smaller than or equal to each case's set membership in the outcome set, across all cases.

truth table. Truth tables are the *indispensable* analytic device used in ***qualitative comparative analysis.*** They are used in logic and with Boolean functions. A truth table has one column for each ***condition*** set used in the analysis, plus one column that represents the ***outcome*** set, and one row for each logically possible combination of conditions. A truth table for a specific analysis will have 2^k rows, where k is equal to the number of conditions included in the analysis. Cases are placed into one of the 2^k logically possible combinations of conditions based on each case's condition set membership values. Each row is then classified using the column representing the outcome set as either being ***sufficient*** for the outcome, not sufficient, or a ***logical remainder*** based on the outcome ***set membership values*** of the cases placed within the row. The ***conjunction*** of conditions for each row where the cases consistently demonstrate the ***outcome*** can be interpreted as a statement of ***sufficiency*** and represents a ***primitive expression.*** These primitive expressions are then logically minimized to provide simpler, yet logically consistent statements of ***sufficiency*** referred to as a ***solution***.

Selected Sources for Additional Information

Website

COMPASSS (COMPArative Methods for Systematic cross-caSe analysis) Worldwide Network; 2014. http://www.compasss.org/about.htm

Books

Caramani, D. (2009). Introduction to the comparative method with Boolean algebra. Los Angeles, CA: SAGE.

Ragin, C. C. (1987). The comparative method: moving beyond qualitative and quantitative strategies. Berkeley, CA: University of California Press.

Ragin, C.C. (2008). *Redesigning social inquiry: Fuzzy sets and beyond*. Chicago, IL: University of Chicago Press.

Rihoux, B. T., & Ragin, C. C. (2009). *Configurational comparative methods: Qualitative comparative analysis (QCA) and related techniques*. Thousand Oaks, CA: SAGE.

Schneider, C. Q. & Wagemann, C. (2012). *Set-theoretic methods for the social sciences: A guide to qualitative comparative analysis*. Cambridge, UK: Cambridge University Press.

Journal Publications and Monographs

Kane, H. L., Lewis, M. E., Williams, P. A., & Kahwati, L. C. (2014). Using qualitative comparative analysis to understand and quantify translation and implementation. *Translational Behavioral Medicine*, *4*(2), 201-208.

Ragin, C. C. (1999), Using qualitative comparative analysis to study causal complexity. *Health Services Research*, *34*(5 Pt 2), 1225-1239.

Schneider, C. Q., & Wagemann C. (2010). Standards of good practice in qualitative comparative analysis (QCA) and fuzzy-sets. *Comparative Sociology*, 2010;9:397-418.

Speer, J., & Basurto, X, (2010). Structuring the calibration of qualitative data as sets for qualitative comparative analysis (QCA). Working paper. Retrieved from http://dx.doi.org/10.2139/ssrn.1831606

Thiem, A., & Dusa. A. (2013). Boolean minimization in social science research: A review of current software for qualitative comparative analysis (QCA). *Social Science Computer Review*, *31*(4), 505-521.

Thygeson, N. M., Piekes, D., & Zutshi, A. (2013). Fuzzy-Set Qualitative Comparative Analysis: A Configurational Comparative Method to Identify Multiple Pathways to Improve Patient-Centered Medical Home Models. AHRQ Publication No: 13-0026-EF. Rockville, MD: Agency for Healthcare Research and Quality, U.S. Department of Health and Human Services. Retrieved from http://pcmh.ahrq.gov/page/fuzzy-set-qualitative-comparative-analysis-configurational-comparative-method-identify-multiple

References

1. Ragin, C.C., *Redesigning social inquiry: fuzzy sets and beyond*. 2008, Chicago: University of Chicago Press.

2. von Eye, A.G. and A. Bogat, *Variable-oriented and person-oriented perspectives of analysis: The example of alcohol consumption in adolescence.* Journal of Adolescence, 2006. **29**(6): p. 981-1004.

3. Ragin, C.C., *The comparative method: moving beyond qualitative and quantitative strategies*. 1987, Berkeley: University of California Press. xvii, 185 p.

4. Ragin, C.C., *Using qualitative comparative analysis to study causal complexity.* Health Services Research, 1999. **34**(5 Pt 2): p. 1225-1239.

5. Caramani, D., *Introduction to the comparative method with Boolean algebra*. 2009, Los Angeles: SAGE.

6. Schneider, C. and C. Wagemann, *Set-theoretic methods for the social sciences: a guide to qualitative comparative analysis*. Strategies for social inquiry. 2012, Cambridge: Cambridge University Press.

7. Thiem, A. and A. Dusa, *Boolean minimization in social science research: A review of current software for qualitative comparative analysis (QCA).* Social Science Computer Review, 2013. **31**(4): p. 505-521.

8. compasss.org. *COMPASSS (COMPArative Methods for Systematic cross-caSe analySis) Worldwide Network. Software*. 2014 [cited 2014 November 5]; Available from: http://www.compasss.org/software.htm.
