# Supplementary material for: Using qualitative comparative analysis in a systematic review of a complex intervention
Source: Syst Rev. 2016 May 4;5:82. doi: 10.1186/s13643-016-0256-y (PMC4875617; doi:10.1186/s13643-016-0256-y)
Supplement: Additional file 2: — Completed review analytic framework, key questions, and summary of findings. This file provides the analytic framework, key questions, and a summary of findings from the completed AHRQ systematic review of interventions to improve medication adherence that was used to examine the suitability of using QCA in a review of a complex intervention. (PDF 156 kb) [file 13643_2016_256_MOESM2_ESM.pdf]

Additional File 2

Completed Review Analytic Framework, Key Questions, and Summary of Findings

Using qualitative comparative analysis in a systematic review of a complex intervention

Leila Kahwati, MD MPH[1,] Sara Jacobs, PhD[1], Heather Kane, PhD[1], Megan Lewis, PhD[1,] Meera Viswanathan, PhD[1], Carol E. Golin, MD[2]

[1] RTI International, Research Triangle Park, NC

[2] Departments of Medicine and Health Behavior, University of North Carolina, Chapel Hill, NC

Corresponding Author:
Leila Kahwati, MD MPH
RTI International
3040 E. Cornwallis Rd.
Research Triangle Park, NC 27709
Tel: (919) 541-6629
Fax: (9109) 541-6683
lkahwati@rti.org

This appendix summarizes the key questions, analytic framework and high-level findings from the completed review that we used for application of QCA. The full evidence report citation is as follows:

> Viswanathan M, Golin CE, Jones CD, Ashok M, Blalock S, Wines RC, Coker-Schwimmer EJ, Grodensky CA, Rosen DL, Yuen A, Sista P, Lohr KN. Closing the quality gap: revisiting the state of the science (vol. 4: medication adherence interventions: comparative effectiveness). Evid Rep Technol Assess (Full Rep). 2012 Sep;(208.4):1-685. PubMed PMID: 24422970.

This report was also published as a manuscript length version as follows:

> Viswanathan M, Golin CE, Jones CD, Ashok M, Blalock SJ, Wines RC, Coker-Schwimmer EJ, Rosen DL, Sista P, Lohr KN. Interventions to improve adherence to self-administered medications for chronic diseases in the United States: a systematic review. Ann Intern Med. 2012 Dec 4;157(11):785-95. Review. PubMed PMID: 22964778.

Key Questions

This evidence synthesis had five Key Questions (KQs), three of which had sub-questions. These questions were:

KQ 1a: Among patients with chronic diseases with self-administered medication prescribed by a provider, what is the comparative effectiveness of interventions aimed at patients, providers, systems, and combinations of audiences in improving medication adherence?

KQ 1b: Is improved medication adherence associated with improvement in patient outcomes?

KQ 2a: Among patients with chronic diseases with self-administered medication prescribed by a provider, what is the comparative effectiveness of policy interventions in improving medication adherence?

KQ 2b: Is improved medication adherence associated with improvement in patient outcomes?

KQ 3a: How do medication-adherence intervention characteristics (e.g., mode of delivery, intervention target, intensity) vary?

KQ 3b: To what extent do the effects of adherence interventions vary based upon their characteristics?

KQ 4: To what extent do the effects of adherence interventions vary based on differences in vulnerable populations?

KQ 5: What unintended consequences are associated with interventions to improve medication adherence?

Analytic Framework

The analytic framework for the completed review is reprinted below (Figure 1 of the Evidence Report). Application of QCA was limited to KQ1a and KQ3.

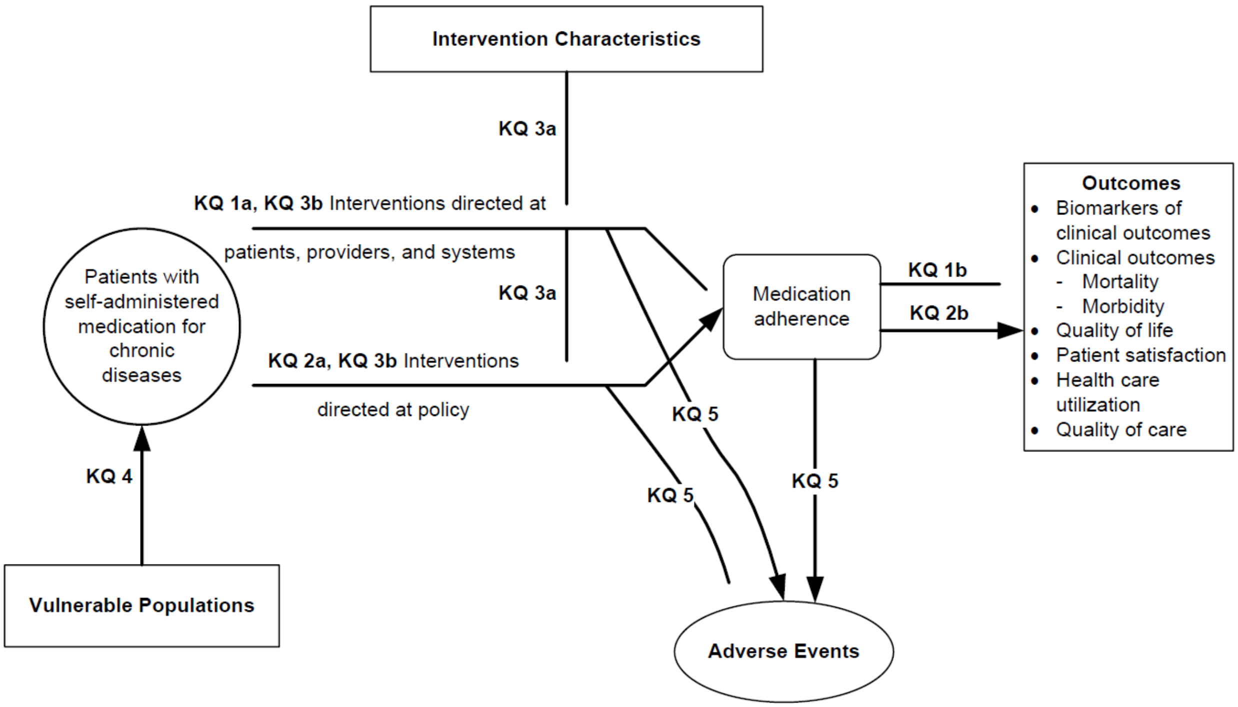

Abbreviation: KQ = Key Question.

Summary of Findings from Completed Review

- 67 eligible studies (61 trials and 4 observational studies)
- Studies included populations with diabetes, hyperlipidemia, hypertension, heart failure, myocardial infarction, asthma, depression, glaucoma, multiple sclerosis, musculoskeletal diseases, and multiple chronic conditions.
- Sixty-two trials of patient, provider, or systems interventions (KQ 1) evaluated 20 different types of interventions;
- The most consistent evidence of improvement in medication adherence across clinical conditions was for interventions that included case management, and educational interventions.
- Within clinical conditions, the strongest support was found for self-management of medications for short-term improvement in adherence for asthma patients; collaborative care or case management programs for short-term improvement of adherence and to improve symptoms for patients taking depression medications; and pharmacist led approaches for hypertensive patients to improve systolic blood pressure.
